# Supplementary material for: Production, reproduction and some adaptation characteristics of Boran cattle breed under changing climate: A systematic review and meta-analysis
Source: PLoS One. 2021 May 28;16(5):e0244836. doi: 10.1371/journal.pone.0244836 (PMC8162631; doi:10.1371/journal.pone.0244836)
Supplement: S1 Checklist — (DOC) [file pone.0244836.s001.doc]

| **Section/topic** | **#** | **Checklist item** | **Reported on page #** |
| --- | --- | --- | --- |
| **TITLE** | | |  |
| Title | 1 | Production, Reproduction and Some Adaptation characteristics of Boran cattle breed under changing climate: A systematic review and meta-analysis | 1 |
| **ABSTRACT** | | |  |
| Structured summary | 2 | **Introduction:** *Climate change affects livestock production and productivity, which could threaten livestock-based food security in pastoral and agro-pastoral production systems of the tropics and sub-tropics. Boran cattle breed is one of the hardiest Zebu cattle reared by Borana Oromo pastoralists for milk and meat production.* *However, there is limited compressive information on production, reproduction and adaption traits of the Boran cattle in Ethiopia. Thus, this paper aims to compile the main production, reproduction and some adaptation traits of Boran cattle based on systematic review and meta-analysis of peer reviewed published and unpublished literature.*  ***Methodology:*** *A combination of systematic review and meta-analysis based on PRISMA guideline was employed. Accordingly, out of 646 recorded articles identified through database searching, 64 were found to be eligible for production, reproduction and adaptation characteristics of the Boran cattle, 28 articles were included in qualitative systematic review while 36 articles were used for quantitative meta-analysis.*  ***Result*:** *The Boran cattle breed has the ability to survive, produce and reproduce under high ambient temperature, utilize low quality forage resources, and resist water shortage or long watering intervals and tick infestations. The review revealed that the breed employs various adaptation responses (morphological, physiological, biochemical, metabolic, cellular and molecular responses) to cope with harsh environmental conditions including climate change, rangeland degradation, seasonal feed and water shortages and high incidences of tick infestations. The meta-analysis using a random-effects model allowed provision of pooled estimates of heritability and genetic correlations for reproduction and production traits, which could be used to solve genetic prediction equations under a population level in purebred Boran cattle. In addition, heritability and genetic-correlation estimates found in the present study suggest that there is high genetic variability for most traits in Boran cattle, and that genetic progress is possible for all studied traits in this breed.*  **Conclusion***: The Boran cattle breed has the ability to survive, produce and reproduce under high ambient temperature, utilize low quality forage resources, and resist water shortage or long watering intervals and tick infestations. However, currently there are several challenges such as recurrent droughts, pasture deterioration and lack of systematic selection and breeding programs that play to undermine the realization of the potential of the breed. Thus, we recommend systematic selection for enhancing the reproductive and production performances without compromising the adaptation traits of the breed coupled with improved management of rangelands.* | 1-2 |
| **INTRODUCTION** | | |  |
| Rationale | 3 | Diffused published literatures are available on production, reproduction and adaptation traits of Boran cattle of Ethiopia under climate changes which requires evidence-based revision to generate comprehensive information for breed conservation and improvement program at community level. This is the core relevance of the review. | 2-3 |
| Objectives | 4 | *This paper aims to compile the main production, reproduction and some adaptation traits of Boran cattle based on systematic review and meta-analysis of peer reviewed and published articles. Unpublished literatures and reports on the subject* | 3 |
| **METHODS** | | |  |
| Protocol and registration | 5 | *The review and meta-analysis protocol was based on the PRISMA guideline given by Moher D, Liberati A, Tetzlaff J, Altman DG, The PRISMA Group (2009). Preferred Reporting Items for Systematic Reviews and Meta-Analyses: The PRISMA Statement. PLoS Med 6(7): e1000097. doi:10.1371/journal.pmed1000097* |  |
| Eligibility criteria | 6 | Both published literatures and thesis reports with full text and published in English, carried out on Boran cattle breed and published before December 15, 2019 were included in the reviews | 4 |
| Information sources | 7 | Data basis from AGORA, SCOPUS, Google Scholar, Google web, PubMed, Science Direct, CAB direct, African Journals online (AJOL) and lists of references of articles from peer reviewed publications. available from 1985 to December 15,2019 were included in the search data base as these are potentially relevant sources information for the reviews. The searches were from June 20 to December 15,2019. | 4-5 |
| Search | 8 | The key words utilized for electronic searches were: ‘growth AND meat production AND Boran cattle’, Milk production AND Boran cattle breed’, ‘Reproduction AND Boran cattle breed and Adaptation AND Boran cattle breed’ | 4 |
| Study selection | 9 | Studies with full text and published in English, carried out on Boran cattle breed and published before December 15, 2019, cross-sectional/ longitudinal/case control studies and relevant response variables of production, reproduction and some adaptation traits were selected for systematic review and meta-analysis | 4-5 |
| Data collection process | 10 | Data were extracted from eligible studies for first author, year of publication, year of study, country, sample size (herd size/records), sampling methods (probability/nonprobability based), breed (Boran, crosses/other zebu breeds) and study traits (production, reproduction and adaptation traits) independently. | 4-5 |
| Data items | 11 | Variables related to production (Meat production, growth, milk production), reproduction and some adaptation traits were collected during data extraction. | 9, 18 |
| Risk of bias in individual studies | 12 | Duplication (articles/data), studies conducted only on cross breeds (to reduce bias/ method related heterogeneity), unpublished organization reports, sample size (*<*50 records in animal studies), and inconsistent data were systematically removed. | 5 |
| Summary measures | 13 | Box plots weighted by the number of records were constructed for each trait to identify potential outliers | 7 |
| Synthesis of results | 14 | The I^2^ index used to quantify the degree of heterogeneity among studies for each trait for each meta-analysis. | 24 |

Page 1 of 2

| **Section/topic** | **#** | **Checklist item** | **Reported on page #** |
| --- | --- | --- | --- |
| Risk of bias across studies | 15 | Studies conducted only on cross breeds (to reduce bias/ method related to heterogeneity), report with sample size (*<*50 records in animal studies) and inconsistent data were systematically removed. | 4 |
| Additional analyses | 16 | Meta-analysis was executed only for traits in which the estimates were based on at least two different databases, to minimize the possible impact of non-independence among articles. For articles in which the standard error for the heritability or correlation estimates were not reported, approximated standard errors were derived by using the combined-variance method. Unpublished literatures and reports were used for systematics review potion | 5-6 |
| **RESULTS** | | |  |
| Study selection | 17 | PRISMA flow diagram was adopted for selection of studies | 4 |
| Study characteristics | 18 | Studies on some Adaptation, production and reproduction characteristics. | 9, 17,18, 19, 26 |
| Risk of bias within studies | 19 | Present data on risk of bias of each study and, if available, any outcome level assessment (see item 12). | 17-19 |
| Results of individual studies | 20 | For all outcomes considered (benefits or harms), present, for each study: (a) simple summary data for each intervention group (b) effect estimates and confidence intervals, ideally with a forest plot. | 17-26 |
| Synthesis of results | 21 | Present results of each meta-analysis done, including confidence intervals and measures of consistency. | 17,18.19.24.27 |
| Risk of bias across studies | 22 | Present results of any assessment of risk of bias across studies (see Item 15). | 20-21 |
| Additional analysis | 23 | Give results of additional analyses, if done (e.g., sensitivity or subgroup analyses, meta-regression, correlation analysis [see Item 16]). | 24,27 |
| **DISCUSSION** | | |  |
| Summary of evidence | 24 | Summarize the main findings including the strength of evidence for each main outcome; consider their relevance to key groups (e.g., healthcare providers, users, and policy makers). | 29 |
| Limitations | 25 | Discuss limitations at study and outcome level (e.g., risk of bias), and at review-level (e.g., incomplete retrieval of identified research, reporting bias). | 29 |
| Conclusions | 26 | Provide a general interpretation of the results in the context of other evidence, and implications for future research. | 29 |
| **FUNDING** | | |  |
| Funding | 27 | The German Academic Exchange Service (DAAD) and the Federal Ministry for Economic Cooperation and Development (BMZ) financed the research under CLIFOOD. | 29 |

*From:*  Moher D, Liberati A, Tetzlaff J, Altman DG, The PRISMA Group (2009). Preferred Reporting Items for Systematic Reviews and Meta-Analyses: The PRISMA Statement. PLoS Med 6(7): e1000097. doi:10.1371/journal.pmed1000097

For more information, visit: **www.prisma-statement.org**.

Page 2 of 2
